# Initial surgery versus conservative management of chronic severe aortic regurgitation in mild symptomatic older patients

**DOI:** 10.1016/j.ijcha.2025.101698

**Published:** 2025-05-14

**Authors:** Mijin Kim, Ha Hye Jo, Sahmin Lee, Byung Joo Sun, Ho Jin Kim, Joon Bum Kim, Sung-Ho Jung, Jong-Min Song, Dae-Hee Kim

**Affiliations:** a Division of Cardiology, Department of Internal Medicine, Pusan National University Hospital, Pusan National University School of Medicine, Busan, Korea; b Division of Cardiology, Asan Medical Center, University of Ulsan College of Medicine, Seoul, Korea; c Division of Cardiology, Department of Internal Medicine, Hanyang Medical Center, Hanyang University College of Medicine, Seoul, Korea; d Department of Cardiothoracic Surgery, Asan Medical Center, University of Ulsan College of Medicine, Seoul, Korea

**Keywords:** Aortic regurgitation, Valve disease surgery, Echocardiography

## Abstract

**Background:**

Aortic valve replacement (AVR) for severe aortic regurgitation (AR) should be weighed against its operative risk. Mortality is lower in patients with mild symptoms than in those with severe symptoms, while the surgical risk remains high in older patients. This study aimed to evaluate the survival benefit of AVR in mildly symptomatic older patients with severe AR.

**Methods:**

From 1996 to 2016, we evaluated 127 older patients with severe AR and mild symptoms. We compared all-cause and cardiac mortality between patients who underwent AVR (OP group, n = 35) and those who were conservatively managed (CONV group, n = 92).

**Results:**

Although patients in the OP group were younger than those in the CONV group (74.2 ± 3.2 vs. 77.3 ± 5.2, p = 0.003), no differences were observed in the Society of Thoracic Surgeons score (1.93 ± 0.95 vs. 2.51 ± 1.8, p = 0.12), comorbidity, indexed left ventricular dimensions (LVEDDi: 41.3 ± 4.4 vs. 41.6 ± 5.9 mm/m^2^, p = 0.80; LVESDi 30.7 ± 4.6 vs. 30.6 ± 5.2 mm/m^2^, p = 0.90), and ejection fraction (46.7 ± 9.9 % vs. 46.9 ± 7.9 %, p = 0.89). Over a median follow-up of 4.2 years, the OP group had significantly lower all-cause (22.9 % vs. 62.0 %, p = 0.010) and cardiac mortality (8.6 % vs. 33.7 %, p = 0.019). In multivariate Cox analysis, AVR remained independently associated with reduced all-cause (HR 0.41; 95 % CI 0.19–0.90; p = 0.027) and cardiac mortality (HR 0.29; 95 % CI 0.09–0.99; p = 0.048).

**Conclusion:**

In mildly symptomatic older patients with severe AR, AVR significantly reduced all-cause and cardiac mortality and should not be withheld solely due to age.

## 1 Introduction

The prevalence of aortic valvular disease increases significantly with age, primarily due to degenerative changes [1,2]. Although aortic stenosis is the most common aortic valve disease in older patients, the prevalence of aortic regurgitation (AR) also increases with age. Significant native AR affects between 2.0 % and 2.5 % of patients aged 70–83 years [2,3].

Classically, aortic valve replacement (AVR) is the gold standard treatment even among older patients despite its risks in this age group. However, AVR for AR in the older patients requires careful evaluation due to numerous concomitant comorbidities and decreased life expectancy associated with aging. Furthermore, the impact on quality of life after surgery should be considered a relevant aspect. Therefore, surgery for severe AR in older adults necessitates a thorough assessment of the risks versus benefits for each individual.

The most recent European Society of Cardiology (ESC) and American Heart Association (AHA)/American College of Cardiology (ACC) guidelines consider symptomatic severe AR a class I indication for surgery, regardless of left ventricular (LV) systolic function. Additionally, surgery is recommended for asymptomatic patients with a left ventricular end-systolic dimension (LVESD) >50 mm, an indexed LVESD >25 mm/m^2^ body surface area (BSA), or a resting left ventricular ejection fraction (LVEF) <50–55 % [4,5]. However, in mildly symptomatic older patients with severe AR, it may be difficult to distinguish whether the symptom is related to aging or has a true cardiac origin. Additionally, although earlier, preemptive AVR is recommended for selected asymptomatic patients, the watchful waiting strategy is still considered a management option for octogenarian patients with asymptomatic severe AR.

While the mortality rate is lower in patients with mild symptoms than those with severe symptoms, older patients still face high surgical risks [6]. In older patients with no or mild symptoms who are candidates for surgery, the decision-making process for AVR remains a clinical dilemma because clinical outcomes after surgical treatment are significantly influenced by preoperative comorbidities and age [[6], [7], [8]]. Therefore, this study aimed to evaluate the survival benefit of surgery in older patients (≥70-years-old) with severe AR and mild symptoms.

## 2 Methods

### 2.1 Patient population

A total of 481 consecutive patients with severe AR aged 70 years and older, from the Asan Medical Center registry in South Korea, were retrospectively reviewed between 1996 and 2016. Among them, 180 patients were excluded due to (1) both preserved LV systolic function (LVEF > 50 %) and only mild-to-moderate LV dilatation (LVEDD < 65 mm and LVESD < 50 mm), (2) aortic valve peak velocity > 3.0 m/sec, or (3) the presence of a prosthetic aortic valve. Consequently, 301 patients who met surgical guideline criteria-LVEF ≤ 50 %, or LVESD ≥ 50 mm, or LVEDD ≥ 65 mm-were included in the final analysis.

**Fig. 1:**
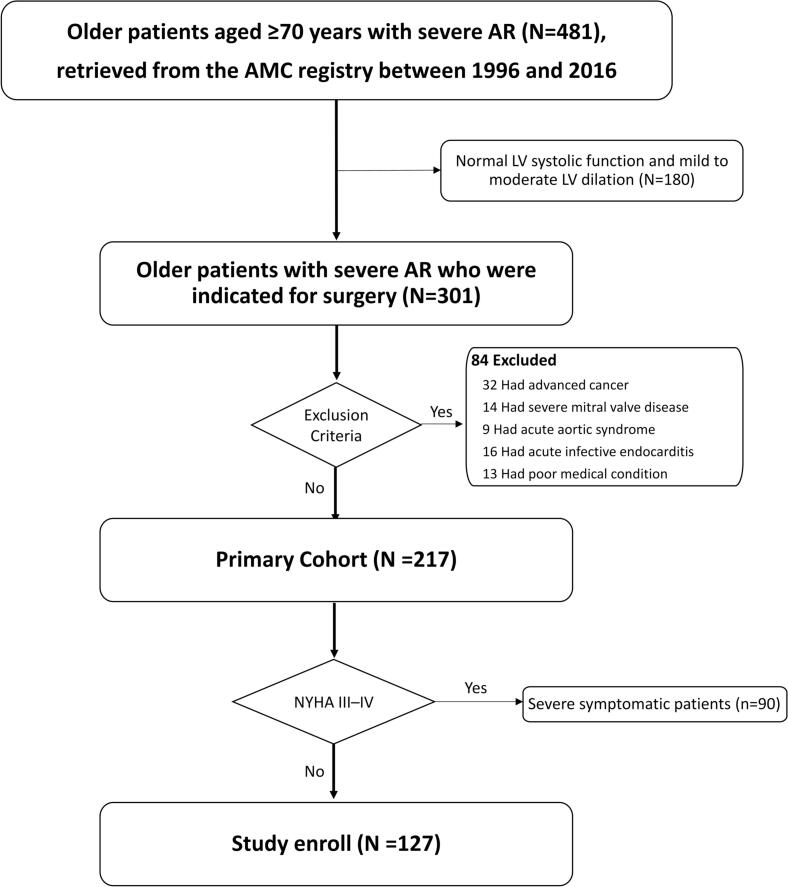
Flowchart for patient selection.

The exclusion criteria for the study were as follows: (1) primary severe mitral valvular diseases, (2) acute aortic syndrome, (3) acute infective endocarditis, (4) serious comorbid conditions such as advanced malignancy with an expected survival of less than one year and (5) patients with New York Heart Association [NYHA] functional class III–IV at the time of diagnosis. As a result, we evaluated a total of 127 older patients (77 men, 76.5 ± 4.9 years) with severe AR and mild symptoms (NYHA functional class I–II at the time of diagnosis). The flowchart for patient selection is summarized in Fig. 1. In addition, a Venn diagram (Supplementary Fig. 1) illustrating the overlap of the three echocardiographic surgical indication criteria (LVEF ≤ 50 %, LVESD ≥ 50 mm, or LVEDD ≥ 65 mm) among the final 127 patients is provided to clarify the distribution of inclusion parameters. Among the 127 patients, 35 underwent AVR within 6 months of diagnosis (OP group) and 92 were conservatively managed (CONV group). The Institutional Review Board of our hospital approved the study protocol, and patients’ informed consent was waived due to the retrospective nature of this study, using general clinical practice. Our investigations were conducted in accordance with the Declaration of Helsinki.

### 2.2 Clinical and echocardiographic data

The baseline clinical and echocardiographic characteristics were retrieved from medical records. Baseline characteristics included age, sex, body mass index (BMI), BSA, underlying comorbidities, heart rhythm, and the Society of Thoracic Surgeons Predicted Risk of Mortality (STS-PROM) score.

All patients in this study underwent complete two-dimensional, M-mode, and Doppler transthoracic echocardiographic examinations. Baseline echocardiography was collected at the time of diagnosis of severe AR. The severity of AR was assessed using an integrated approach that included color Doppler measurement of jet width in the LV outflow tract, measurement of the vena contracta width, the percentage of the jet width relative to the LV outflow tract width, the presence of diastolic flow reversal in the descending aorta, and evaluation of the regurgitant volume and fraction [9]. LVEDD and LVESD were measured by M-mode or 2-dimensional echocardiography at rest according to guidelines [10]. LVEF was determined using the modified Simpson’s method from the apical views. Left atrial (LA) volume was assessed using the same method at end-systole. Tricuspid regurgitation velocity was determined by using continuous wave Doppler echocardiography.

### 2.3 Surgical procedure

In the initial surgery group, isolated AVR and AVR with aortic root replacement were successfully performed in 17 (49 %) and 12 (34 %) patients, respectively. Concomitant mitral valve repair or replacement at the time of AV surgery was performed in 6 (17 %) patients due to moderate mitral regurgitation (MR). Additionally, concomitant coronary artery bypass grafting was performed on 4 patients (14 %).

The surgical procedure was executed through a median sternotomy or minimally invasive incision under general anesthesia and cardiopulmonary bypass. After valve sizing, a tissue valve was selected and implanted based on anatomical considerations and the surgeon’s preference. The surgical result was immediately evaluated using intraoperative transesophageal echocardiography.

### 2.4 Outcomes and follow-up

The primary endpoint of the study was all-cause mortality, including operative mortality. The median duration of follow-up was 4.2 years [Interquartile range (IQR) 2.3–7.8 years]. Mortality data were obtained from our medical records and the National Death Index. Additionally, deaths were classified as either cardiac or non-cardiac in nature.

### 2.5 Statistical analysis

Continuous variables, presented as means with standard deviations, were compared using Student’s *t*-test or the Wilcoxon rank-sum test, depending on their distribution. Categorical and ordinal variables, presented as frequencies and percentages, were compared using the χ2 or Fisher’s exact test, as appropriate.

The event-free survival rate was estimated through Kaplan–Meier analysis, and differences between groups were compared with the log-rank test. To investigate the relative risk associated with different treatments (OP vs. CONV), Cox proportional hazards models of overall death and cardiac death were used, and the results were described by their estimated hazard ratios (HR) and 95 % confidence intervals (CI). For overall death and cardiac death, after unadjusted analyses were initially performed, multivariable Cox regression analyses were conducted to sequentially adjust for potentially confounding covariates. We considered variables with p-values less than 0.10 in the baseline characteristics between the surgical group and the CONV group as potential confounding covariates. We started with a model that adjusted for patient age, sex, the STS score, atrial fibrillation, indexed LV end-diastolic dimension, indexed LV end-systolic dimension, LA size, and LVEF. Multicollinearity among covariates was assessed using variance inflation factors (VIFs). All VIFs were below 5, indicating no significant collinearity among the included echocardiographic parameters. Additionally, univariate Cox regression analyses were performed in the overall population to identify variables associated with overall and cardiac mortality.

All reported p-values are two-sided, and a p-value < 0.05 was considered statistically significant. All statistical analyses were performed using SAS software, version 9.4 (SAS Institute, Cary, North Carolina) and R software version 4.0.3 (R Foundation for Statistical Computing, Vienna, Austria; https://www.r-project.org).

## 3 Results

### 3.1 Baseline patient characteristics

Baseline patient characteristics are summarized in Table 1. Compared to the CONV group, patients in the OP group were younger (74.2 ± 3.2 vs. 77.3 ± 5.2 years, p = 0.003), and had higher BMI (23.8 ± 2.5 vs. 22.4 ± 3.0 kg/m^2^, p = 0.02) and BSA (1.7 ± 0.2 vs. 1.6 ± 0.2 m^2^, p < 0.001). No significant differences were observed in sex distribution or STS scores (1.93 ± 0.95 vs. 2.51 ± 1.8, p = 0.12). Furthermore, there were no significant differences between the two groups in terms of past comorbidities. Evaluation of the baseline echocardiographic characteristics revealed that the OP group exhibited larger LA and LV dimensions than those of the CONV group. However, the indexed LV dimensions (LVEDDi: 41.3 ± 4.4 vs. 41.6 ± 5.9 mm/m^2^, p = 80; LVESDi 30.7 ± 4.6 vs. 30.6 ± 5.2 mm/m^2^, p = 0.90) and LVEF (47 ± 8 % vs. 47 ± 10 %, p = 0.89) were similar between the two groups. Similarly, the vena contracta width of AR did not differ significantly between the two groups. The etiology of AR was aortic root dilatation in 61 (48 %) and isolated AR in 66 (51.9 %) of the overall patients. In the surgical group, surgical procedures performed included isolated AVR or aortic valvuloplasty (n = 13), combined root replacement (n = 12), mitral annuloplasty (n = 4), mitral valve replacement (n = 2), tricuspid annuloplasty (n = 5), and maze operation (n = 7). Concomitant bypass surgery was performed in 4 patients.

**Table 1:** Baseline characteristics between medical treatment group and surgical treatment group.

Characteristics	Overall (N = 127)	Treatment option		p-value^a^
		Medical treatment (N = 92)	Surgical treatment (N = 35)	
Clinical data				
Age	76.5 ± 4.9	77.3 ± 5.2	74.2 ± 3.2	**0.003**
Gender, n (%)				0.35
Female	50 (39.4 %)	39(42.4 %)	11(31.4 %)	
Male	77 (60.6 %)	53(57.6 %)	24(68.6 %)	
Body mass index (kg/m^b^)	22.8 ± 2.9	22.4 ± 3.0	23.8 ± 2.5	0.02
Body surface area (m^b^)	1.6 ± 0.2	1.6 ± 0.2	1.7 ± 0.2	**<0.001**
STS score	2.35 ± 1.63	2.51 ± 1.81	1.93 ± 0.95	0.12
NYHA classification				0.14
NYHA Functional class I	103(81.1 %)	78 (84.8 %)	25 (71.4 %)	
NYHA Functional class II	24(18.9 %)	14(15.2 %)	10(28.6 %)	

Medical history				
Atrial fibrillation, n (%)	36 (28.3 %)	26 (28.3 %)	10 (28.6 %)	>0.9
Hypertension, n (%)	66 (52.0 %)	48 (52.2 %)	18 (51.4 %)	>0.9
DM, n (%)	10 (7.9 %)	7 (7.6 %)	3 (8.6 %)	>0.9
Previous Coronary artery disease^b^, n (%)	17 (13.4 %)	13(14.1 %)	4 (11.4 %)	0.91
Chronic obstructive pulmonary disease, n (%)	4 (3.1 %)	2 (2.2 %)	2 (5.7 %)	0.65
Cerebrovascular accident, n (%)	12 (9.4 %)	10(11 %)	2 (5.7 %)	0.58
Chronic kidney disease, n (%)	9 (7.1 %)	9 (9.8 %)	0 (0 %)	0.13
Previous myocardial infarction, n (%)	4 (3.1 %)	3 (3.3 %)	1 (2.9 %)	>0.9
Malignancy, n (%)	9 (7.1 %)	5 (5.4 %)	4 (11.4 %)	0.43

Echocardiography finding				
Left ventricular end diastolic dimension (mm)	66.8 ± 6.8	65.7 ± 6.3	69.5 ± 7.1	**0.004**
Left ventricular end systolic dimension (mm)	48.9 ± 6.3	48.0 ± 5.7	51.4 ± 7.0	**0.005**
Indexed left ventricular end diastolic dimension (mm/m^2^)	41.5 ± 5.5	41.6 ± 5.9	41.3 ± 4.4	0.80
Indexed left ventricular end systolic dimension (mm/m^2^)	30.6 ± 5.0	30.6 ± 5.2	30.7 ± 4.6	0.90
Left ventricular ejection fraction (%)	46.7 ± 9.3	46.7 ± 9.9	46.9 ± 7.9	0.89
Left atrial size (mm)	44.1 ± 8.3	43.3 ± 8.6	46.3 ± 7.2	0.07
Peak TR velocity	2.7 ± 0.4	2.7 ± 0.4	2.6 ± 0.5	0.29
MR severity				0.34
None/Trivial	31 (24.4 %)	22 (23.9 %)	9 (25.7 %)	
Mild	72 (56.7 %)	54 (58.7 %)	18 (51.4 %)	
Moderate	20 (15.7 %)	12 (13.0 %)	8 (22.9 %)	
Moderate to severe	4 (3.1 %)	4 (4.3 %)	0 (0.0 %)	
Etiology of AR				0.39
Quadricuspid aortic valve	1 (0.8 %)	1 (1.1 %)	0 (0.0 %)	
Isolated aortic root dilatation^c^	20 (15.8 %)	14 (15.2 %)	6 (17.2 %)	
Isolated prolapse or flail	26 (20.5 %)	16 (17.4 %)	10 (28.6 %)	
Isolated restriction or retraction	40 (31.5 %)	31 (33.7 %)	9 (25.7 %)	
Mixed mechanism	40 (31.5 %)	30 (32.6 %)	10 (28.6 %)	
Vena contracta width of aortic regurgitation (mm)	8.0 ± 1.6	8.0 ± 1.7	8.0 ± 1.5	0.96

AR, Aortic regurgitation; DM, diabetes mellitus; LVOT, left ventricular outflow tract; NYHA, New York Heart Association; MR, Mitral regrigutation; STS score, The Society of Thoracic Surgeons score; TR, tricuspid regurgitation.

a Wilcoxon rank sum test; Pearson's Chi-squared test; Fisher's exact test.

b Coronary artery disease was defined as a history of percutaneous coronary intervention. In the OP group, patients who underwent coronary artery bypass grafting (CABG) during aortic valve surgery were also classified as having CAD.

c Aortic root dilatation was defined as an aortic root diameter >40 mm.

### 3.2 Comparison of outcomes between the OP and CONV groups

Event rates in both groups over a period of up to 10 years are presented in Table 2. The median follow-up duration was 4.6 years (IQR 2.5–6.8 years) in the OP group and 3.8 years (IQR 1.9–8.1 years) in the CONV group. In the OP group, the median interval between baseline echocardiography and aortic valve surgery was 18 days (IQR, 6.5–43 days). At the end of the follow-up period, 8 patients (22.9 %) in the OP group had died. Among them, 5 patients died of non-cardiac causes while 3 patients died of cardiac causes. The causes of the 3 cardiac deaths in the OP group included operative mortality (n = 1), aortic valve dysfunction (n = 1), and ischemic heart disease (n = 1). At the end of the follow-up, 57 patients (62.0 %) in the CONV group had died. Among them, 26 patients died of non-cardiac causes (28.3 %), and 31 patients (33.7 %) died of cardiac causes. The causes of the 31 cardiac deaths in the CONV group included heart failure in 13 patients, ischemic heart disease in 11, and sudden cardiac death in 7. Only 2 (2.2 %) patients in the CONV group underwent surgery during the follow-up. The surgical procedures included 1 isolated AVR and 1 AVR with mediastinal mass excision.

**Table 2:** Clinical outcomes between medical treatment group and surgical treatment group

Clinical outcomes	Overall (N = 127)	Treatment option		p-value*^1^*
		Medical treatment (N = 92)	Surgical treatment (N = 35)	
All cause death	65 (51.2 %)	57 (62.0 %)	8 (22.9 %)	**0.010**
Cardiac death	34 (26.8 %)	31(33.7 %)	3 (8.6 %)	**0.019**
Non-cardiac death	31(24.4 %)	26(28.3 %)	5(14.3 %)	0.20
30-days operative mortality	1 (0.8 %)	0 (0 %)	1(2.9 %)	NA
Reoperation of aortic valve	3 (2.4 %)	0 (0 %)	3(8.6 %)	NA

Median follow up duration 4.2 years (Interquartile range [IQR] 2.3–7.8).

NA; Not Applicable.

The OP group had a significantly lower risk of overall death (HR 0.39; 95 % CI 0.18 to 0.82; p = 0.013) and cardiac death (HR 0.27; 95 % CI 0.08 to 0.88; p = 0.029)*.* In multivariable analysis adjusting for age, sex, STS score, atrial fibrillation, indexed LVEDD, LVESD, LA size, and LVEF, AVR remained independently associated with reduced mortality (Table 3).”.

**Table 3:** Hazard Ratios of operation^a^.

Model	Overall death		Cardiac death	
	HR (95 % CI)	p-value	HR (95 % CI)	p-value
Univariate	0.39 (0.18–0.82)	0.013	0.27 (0.08–0.88)	0.029
Multivariate*	0.41 (0.19–0.90)	0.027	0.29 (0.09–0.99)	0.048

a Operation refers to aortic valve surgery, which was modeled as a time-dependent covariate in multivariable Cox analysis.

* Adjusted for age, sex, STS score, atrial fibrillation, indexed LV end-diastolic dimension, indexed LV end-systolic dimension, LA size, and LVEF.

**Fig. 2:**
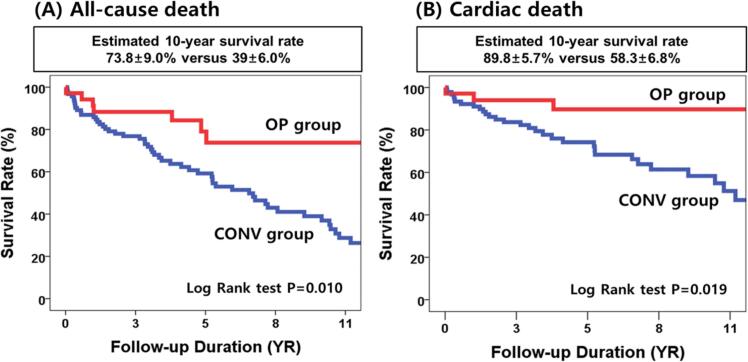
Kaplan–Meier curves for event-free survival rates of overall death (A) and cardiac death (B).

**Fig. 3:**
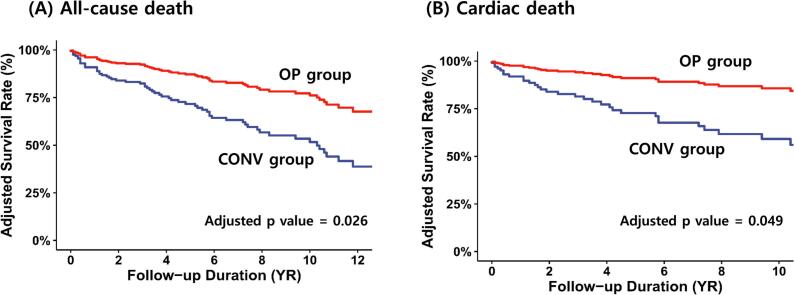
Adjusted Kaplan–Meier curves for event-free survival rates of overall death (A) and cardiac death (B). Adjustment was performed for age, sex, STS score, atrial fibrillation, indexed LVEDD and LVESD, LVEF, and LA size.

The Kaplan–Meier curves for event-free survival rates of overall and cardiac death are presented in Fig. 2. The estimated 10-year overall survival rates of the OP and CONV groups were 73.8 ± 9.0 % and 39.0 ± 6.0 %, respectively (Fig. 2A) (log-rank p = 0.010). Furthermore, the Kaplan–Meier analysis estimated the 10-year event-free survival rate of cardiac death at 89.8 ± 5.7 % for patients in the OP group and 58.3 ± 6.8 % for patients in the CONV group (Fig. 2B) (log-rank p = 0.019). Fig. 3 shows the adjusted Kaplan–Meier curves for event-free survival from overall (Fig. 3A) and cardiac death (Fig. 3B), estimated using time-dependent Cox models with adjustment for age, sex, STS score, atrial fibrillation, indexed LV dimensions, LVEF, and LA size. Even after multivariable adjustment, surgery remained significantly associated with lower risks of overall and cardiac mortality compared to conservative management.

To assess the temporal consistency of treatment effects, patients were stratified into two cohorts based on enrollment period: 1996–2006 and 2007–2016. Subgroup analyses revealed no significant interaction between treatment strategy and risk of all-cause mortality (p for interaction = 0.54) or cardiac mortality (p for interaction = 0.71), indicating that the survival benefit associated with AVR remained stable over time.

### 3.3 Predictors of clinical events

Univariate Cox regression analysis was used to evaluate predictors of all-cause and cardiac death in the overall patient cohort (Table 4). For both overall and cardiac mortality, indexed LVEDD and aortic valve surgery were identified as significant predictors in the univariate analysis. In a subsequent multivariable Cox regression model for overall mortality, aortic valve surgery remained the only significant predictor. In contrast, for cardiac mortality, both indexed LVEDD and aortic valve surgery remained independently associated with outcome.

**Table 4:** Predictors of clinical events – Univariate and multivariate cox regression analysis.

Variable	All-cause Death				Cardiac Death			
	Univariate		Multivariate		Univariate		Multivariate	
	HR (95 % CI)	p-value	HR (95 % CI)	p-value	HR (95 % CI)	p-value	HR (95 % CI)	p-value
Age, per 10 years	1.47 (0.91–2.39)	0.117			1.03 (0.96–1.10)	0.413		
Sex	0.91 (0.56–1.49)	0.709			0.96 (0.48–1.91)	0.909		
Diabetes	1.30 (0.47–3.60)	0.615			0.91 (0.50–1.65)	0.758		
Previous coronary artery disease	1.56 (0.77–3.18)	0.219			1.90 (0.78–4.61)	0.155		
Atrial fibrillation	1.27 (0.75–2.16)	0.379			0.91 (0.41–2.01)	0.808		
STS score	1.13 (0.99–1.28)	0.062			1.00 (0.80–1.26)	0.972		
Indexed left ventricular end diastolic dimension	1.05 (0.96–1.01)	**0.046**	1.04 (1.00–1.09)	0.072	1.14 (1.03–1.26)	**0.011**	1.07 (1.01–1.13)	**0.021**
Indexed left ventricular end systolic dimension	1.03 (0.98–1.08)	0.203			0.93 (0.84–1.04)	0.205		
LV ejection fraction, per 1 %	1.00 (0.98–1.03)	0.821			1.02 (0.98–1.06)	0.276		
Significant MR	1.69 (0.88–3.25)	0.117			2.06 (0.90–4.71)	0.086		
Aortic valve operation^1^	0.39 (0.19–0.82)	**0.013**	0.40 (0.19–0.85)	**0.018**	0.27 (0.08–0.88)	**0.030**	0.29 (0.09–0.94)	**0.040**

^1^Modeled as a time-dependent covariate.

## 4 Discussion

In this study, we evaluate clinical outcomes between older patients with severe AR and mild symptoms treated with surgery and only conservative medical treatment. The major findings of our study can be summarized as follows: first, AV surgery is associated with significant long-term reductions in overall mortality and cardiac mortality in older patients with asymptomatic or mildly symptomatic severe AR compared with conservative management. Second, AVR was independently associated with lower all-cause mortality and cardiac mortality.

The prognosis in patients with severe AR is poor without aortic valve surgery, even if the symptoms at the diagnosis are mild [11]. AV surgery is a definite treatment for severe AR; however, it should be considered only when the benefits of the surgery outweigh its risks. Though the risk of surgery increases with age, operative mortality remains low in patients with mild symptoms at the time of diagnosis [[6], [7], [8]]. Brown et al. previously reported a decline in operative mortality from 3.5 % to 2.4 % between 1997 and 2006 [12]. Supporting this trend, a large-scale UK registry study of 79,173 patients undergoing isolated surgical AVR between 1996 and 2018 demonstrated a reduction in in-hospital or 30-day mortality from 5.28 % to 1.06 % [13]. In addition, single-center studies from the Netherlands and the UK reported reductions from 1.9 % to 0.9 % and 2.9 % to 0.7 %, respectively [14,15]. Similarly, Barreto-Filho et al. revealed that the operative mortality of AVR in patients aged 65 years or older significantly declined from 7.6 % to 4.2 % between 1999 and 2011 [16]. Although outcomes from high-volume centers may not be directly generalizable to smaller institutions, the overall evidence suggests significant improvements in operative mortality for isolated AVR over the past two decades. Due to advances in aortic valve surgery techniques and perioperative care, several studies have reported that the operative mortality of AVR has significantly declined, especially in older patients. In our study, the operative mortality rate of AVR in patients aged 70 years or older with low operative risk was 2.9 %, which is substantially lower than that reported in previous studies. This reduced operative mortality observed in our study compared to those of previous studies may be attributable to patients in our cohort exhibiting lower risk STS-PROM scores and milder symptoms.

Despite the significant recent decrease in operative mortality, factors such as age, life expectancy related to underlying diseases, economic status, comorbidities, and operative risk pose significant barriers in making surgical decisions for the older population. Therefore, a watchful waiting strategy remains a viable management option for octogenarians patients with asymptomatic and mildly symptomatic severe AR. Prior studies have shown that surgical AVR in older adults is an effective and acceptable treatment for aortic valve disease [[17], [18], [19], [20], [21]]. However, until recently, there were no data directly comparing initial surgical treatment with conservative medical therapy in older patients with mildly symptomatic severe AR. Our study demonstrated that surgical risk was acceptable and that overall mortality and cardiac mortality in patients with severe AR and mild symptoms, aged over 70 years, who underwent initial AV surgery were lower than those in the conservative medical treatment group. Therefore, the findings of this study support the survival benefit of the initial surgical strategy in patients over 70 years with severe AR and mild symptoms.

Several prior studies have identified LV dimensions, LVEF, age, and preoperative comorbidities as predictors of overall mortality in patients with severe AR, primarily in post-AVR cohorts [8,22,23]. In contrast, our study assessed all-cause mortality from the time of diagnosis, including patients managed both surgically and conservatively. In this broader context, AVR emerged as the sole independent predictor of overall mortality, while both AVR and indexed LVEDD were independently associated with cardiac mortality. This difference likely reflects the heterogeneity of our study population and the inclusion of non-operated patients. Thus, AVR should be considered a potentially beneficial strategy for improving outcomes in appropriately selected older patients with severe AR.

This study has certain limitations. First, our study was a retrospective observational study subject to potential selection and ascertainment biases. Therefore, we cannot exclude the possibility that unmeasured confounding factors influenced the observed findings. Second, patient age significantly differed between the two groups. However, we adjusted for age, sex, STS score, atrial fibrillation, indexed LVEDD, LVESD, LA size, and LVEF in our multivariable outcome analysis; the other baseline characteristics were similar between the two groups. Third, the number of events per variable in our multivariable model was relatively low, which may raise concerns regarding potential overfitting. covariates were selected based on clinical relevance, and model complexity was minimized to reduce this risk. Finally, despite careful covariate selection based on clinical relevance and assessment for multicollinearity, the limited number of events relative to the number of variables included in the multivariable Cox regression model raises the possibility of overfitting. Although recent studies suggest that lower events-per-variable thresholds may be acceptable in certain contexts, the risk of model instability cannot be entirely excluded. Therefore, our findings should be interpreted with caution and warrant external validation in larger datasets [24,25]. Fourth, although patients with primary severe mitral valve disease were excluded, we included patients with moderate or moderate-to-severe secondary MR. The presence of significant MR may have contributed to increased LV dimensions and acted as a potential confounding factor in the observed association between LV size and cardiovascular mortality. Fifth, although vena contracta width was assessed in all patients, other quantitative echocardiographic parameters such as regurgitant volume, regurgitant fraction, and effective regurgitant orifice area were not routinely evaluated. In addition, advanced markers such as LV end-systolic volume index and global longitudinal strain, which have been shown to have prognostic significance in patients with AR, were not available in this retrospective dataset. These limitations may have restricted a more comprehensive assessment of AR severity and subclinical LV dysfunction. Sixth, this study was conducted at a single center and due to the notably small sample size and limited number of clinical events, it is likely underpowered, significantly affecting its generalizability. Therefore, the results should be interpreted with caution and may not be applicable to all octogenarians with severe AR. Nonetheless, this study offers valuable preliminary insights and highlights the need for larger, multicenter prospective studies to validate these findings.

## 5 Conclusion

Compared with conservative medical treatment, aortic valve surgery is associated with significant decreases in the adjusted risks of overall death and cardiac death in older patients with severe AR and mild symptoms. As a result, aortic valve surgery can be performed in older patients with acceptable outcomes and should not be deferred solely due to older age.

## CRediT authorship contribution statement

**Mijin Kim:** Writing – original draft, Visualization, Methodology, Conceptualization. **Ha Hye Jo:** Methodology, Conceptualization. **Sahmin Lee:** Investigation. **Byung Joo Sun:** Resources, Investigation. **Ho Jin Kim:** Validation, Software. **Joon Bum Kim:** Resources, Data curation. **Sung-Ho Jung:** Resources, Data curation. **Jong-Min Song:** Validation, Supervision, Resources. **Dae-Hee Kim:** Writing – review & editing, Supervision, Methodology, Data curation, Conceptualization.

## Declaration of competing interest

The authors declare that they have no known competing financial interests or personal relationships that could have appeared to influence the work reported in this paper.
