# Supplementary material for: Initial surgery versus conservative management of chronic severe aortic regurgitation in mild symptomatic older patients
Source: Int J Cardiol Heart Vasc. 2025 May 14;59:101698. doi: 10.1016/j.ijcha.2025.101698 (PMC12141872; doi:10.1016/j.ijcha.2025.101698)

SUPPLEMENTAL MATERIAL

The authors have provided this appendix to provide readers with additional information about their work.

**Supplementary Figure 1.** Venn diagram illustrating the overlap of echocardiographic inclusion criteria among older patients with severe AR

**Supplementary Figure 1.** Venn diagram illustrating the overlap of echocardiographic inclusion criteria among older patients with severe AR
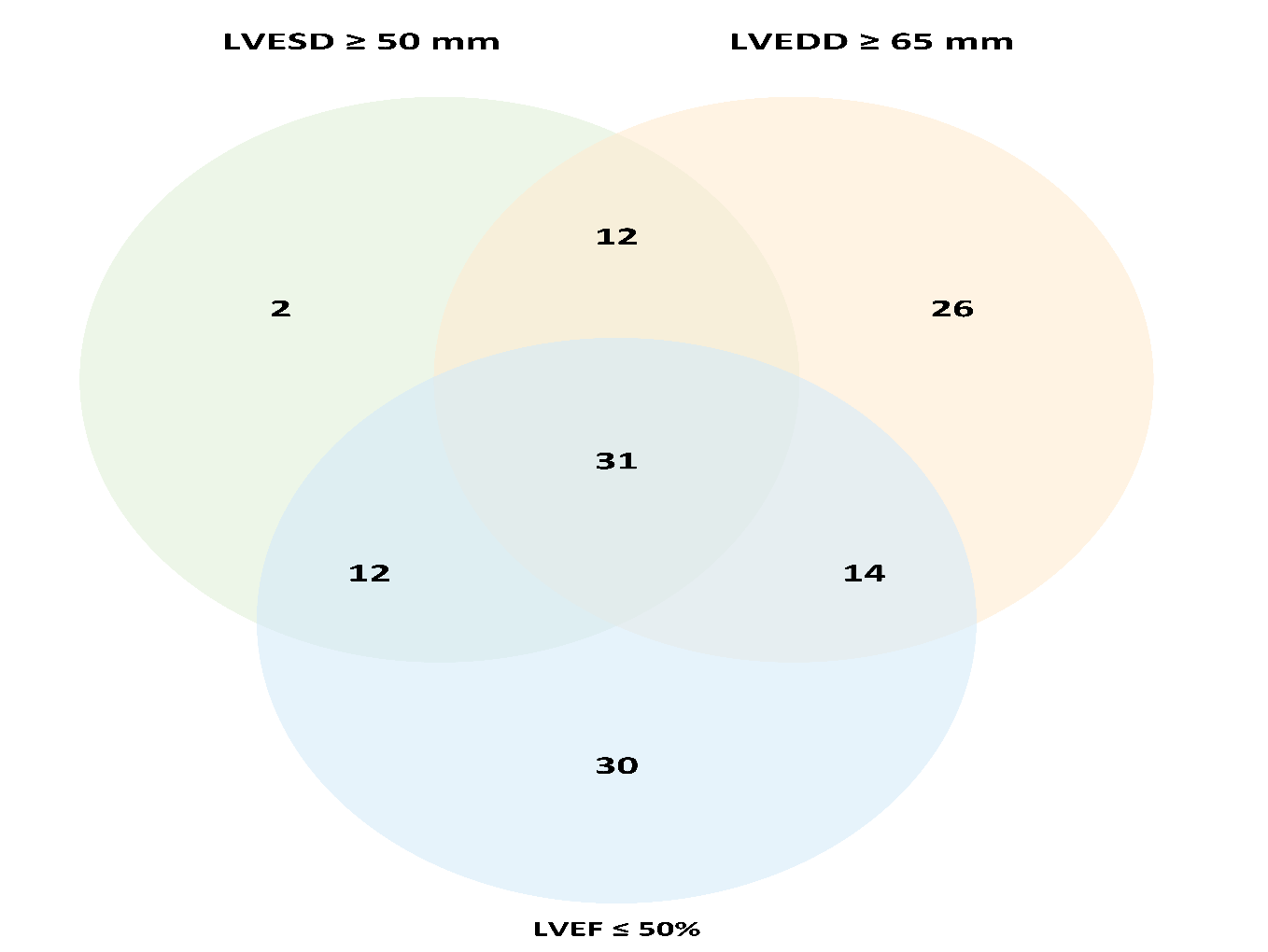

Supplement: Supplementary Data 1 [file mmc1.docx]
