# Supplementary figures and images for: Initial surgery versus conservative management of chronic severe aortic regurgitation in mild symptomatic older patients
Source: Int J Cardiol Heart Vasc. 2025 May 14;59:101698. doi: 10.1016/j.ijcha.2025.101698 (PMC12141872; doi:10.1016/j.ijcha.2025.101698)

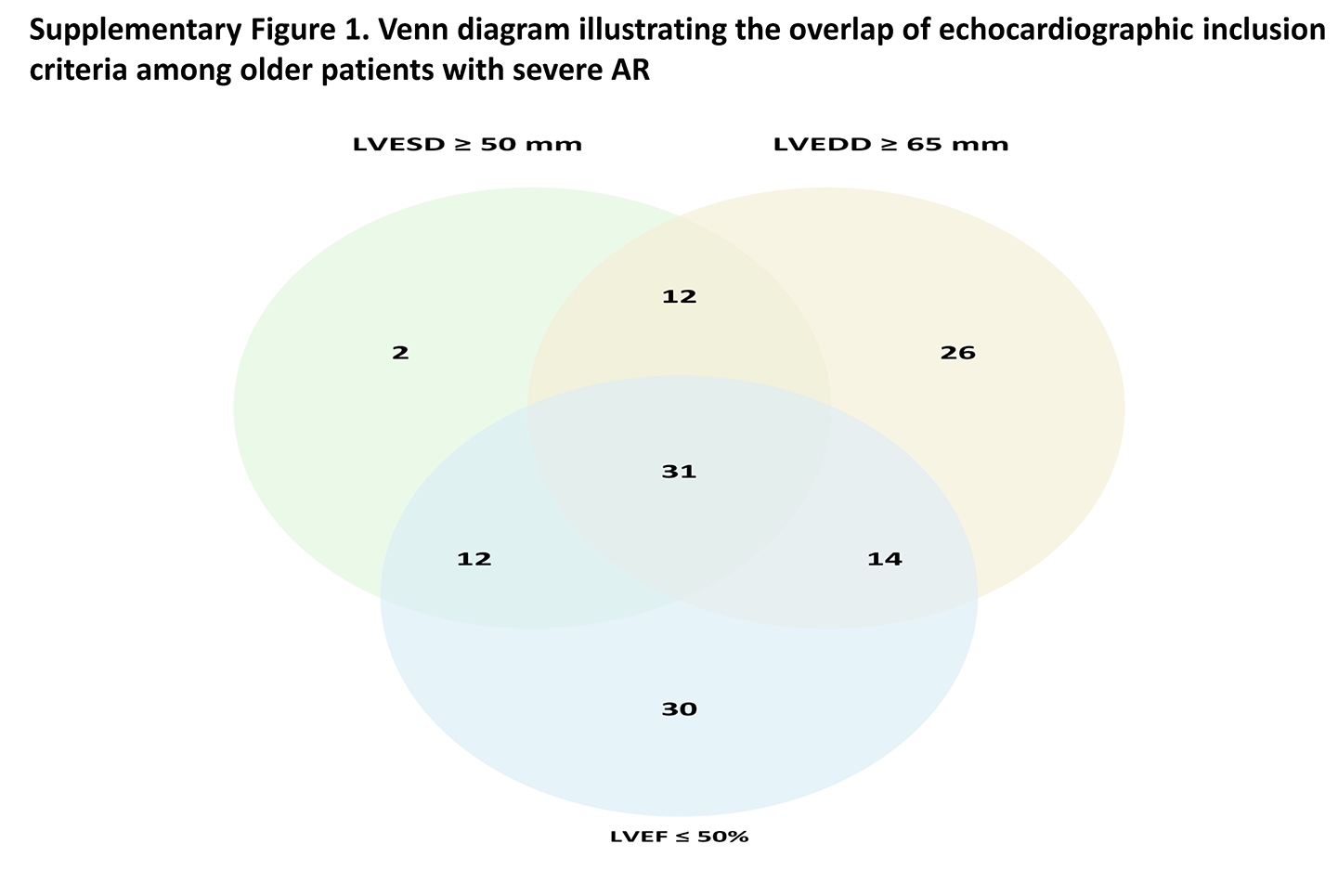

Supplement: Supplementary Figure 2 [file mmc2.jpg]
